# Effect of μPlasma Modification on the Wettability and the Ageing Behaviour of Glass Fibre Reinforced Polyamide 6 (GFPA6)

**DOI:** 10.3390/ma14247721

**Published:** 2021-12-14

**Authors:** Chang Che, Behnam Dashtbozorg, Xiaoying Li, Hanshan Dong, Mike Jenkins

**Affiliations:** School of Metallurgy and Materials, University of Birmingham, Birmingham B15 2TT, UK; cxc852@bham.ac.uk (C.C.); b.dashtbozorg@bham.ac.uk (B.D.); x.li.1@bham.ac.uk (X.L.); h.dong.20@bham.ac.uk (H.D.)

**Keywords:** microplasma, thermoplastic composites, polyamide 6, wettability, ageing, contact angles

## Abstract

Glass fibre reinforced polyamide 6 (GFPA6) thermoplastic composites (TPCs) are promising materials with excellent properties, but due to their low surface free energy they are usually difficult to wet, and therefore, possesses poor adhesion properties. μPlasma modification offers potential solutions to this problem through functionalisation of the GFPA6 surface. In this study, the effect of μPlasma on the wetting behaviour of GFPA6 surfaces was investigated. Following single μPlasma treatment scans of GFPA6 samples, a substantial enhancement in wettability was observed. However, the effect of the μPlasma modification was subject to an ageing (hydrophobic recovery) phenomenon, although the enhancement was still partially maintained after 4 weeks. The ageing process was slower when the GFPA6 material was pre-dried and stored in low humidity conditions, thereby demonstrating the importance of the storage environment to the rate of ageing. Orientation of the fibres to the observed contact angle was found to be crucial for obtaining reproducible measurements with lower deviation. The influence of testing liquid, droplet volume and surface texture on the repeatability of the measured contact angle were also investigated.

## 1. Introduction

Thermoplastic composites (TPCs) are a class of polymers that have attracted much attention due to the desirable combination of good mechanical properties, ease of manufacture, low density and their ease of recycling [1,2]. In recent years, the industrial demand for glass fibre reinforced polyamides has increased, especially in the automotive sector, due to their excellent thermal stability, high toughness and stiffness [3,4,5,6,7]. For example, Polyamide 6 with glass fibre reinforcement offers good thermal stability, and higher tensile strength [1,8], which makes it an attractive material for the automotive industry. Often there is a requirement that the TPC is bonded to other structural materials, such as metals or other composites. However, due to the typically low surface free energy, poor chemical reactivity, polymers and TPCs are usually difficult to wet, and therefore, possess poor adhesion properties [9,10]. A potential solution to this problem is through surface modification of the polymers or TPCs to improve their surface properties, while still retaining their desirable bulk properties.

A number of studies have demonstrated that surface treatments, such as laser treatment, chemical treatment, plasma treatment, ion beam and ion implantation [11,12,13,14,15,16], can enhance the wettability of different materials. In particular, plasma treatment of polymer materials has been shown to modify the surface by removing contaminants, introducing functional groups, and improving the surface free energy without affecting the substrate material [17]. Consequently, plasma surface modification of polymers has become an attractive solution to overcome the challenges associated with the poor wetting and adhesion of these materials. However, plasma-based techniques, such as active screen plasma nitriding, are usually operated at low pressures, and therefore, require costly treatment chambers capable of achieving low pressures coupled with sufficiently powerful vacuum systems necessary to reach the desired pressures [17,18,19]. In addition, it is difficult, if not impossible, to achieve continuous treatment using the active screen plasma technique. To overcome these limitations, atmospheric plasma treatments have been explored, an example is a μPlasma modification. These plasma treatment techniques offer the ability to perform localised treatments under atmospheric conditions and thus demonstrate great promise for future industrial uptake [20,21,22].

Numerous studies have demonstrated that plasma surface treatment can modify the wettability of polymer surfaces significantly [23,24,25,26,27,28], however, despite its great potential, only two publications reported μPlasma modification on polymers [20,29]. Plasma surface treatment on different kinds of polymers has also been reported, including polyamide materials, such as polyamide 12, polyamide 6,6 and polyamide 6 [30,31,32,33,34], however, there are no studies that report on the plasma treatment of glass-fibre reinforced polyamide 6.

While plasma treatments are effective in improving polymer surface wettability, it is well established that these enhancements are not permanent [35,36]. A pronounced increase in wettability is typically observed immediately following plasma treatment, with a gradual decrease over time, to the point where the wettability of the surface almost matches the untreated polymer [10,37]. This decay of the wettability over time is known as the hydrophobic recovery or ageing of the surface [38], and studies have reported these phenomena with different forms of plasma treatments, including low-pressure plasma treatment of polyamide 6 rods [10] and polyamide 6 sheets [39], NH_3_ plasma treatment of polyamide 6 foil [40], and atmospheric pressure plasma treatment of polyamide 6 fibres [41]. However, the ageing data was typically reported on a daily or weekly basis, with minimal focus on the initial changes within the first few minutes or hours after the treatment. Therefore, given the dynamic nature of the surface following plasma treatment, it is critical to investigate and characterise the initial wettability fluctuations and ageing behaviour following plasma treatment to enable a better understanding of this phenomenon, as well as approaches to prevent or slow this ageing behaviour.

To characterise the change in surface wettability after plasma treatments, many studies rely on the measurement of the contact angle. However, the reliability and validity of these measurements on hydrophilic, and potentially rough, surfaces have proven to be challenging. The measurement of contact angle is affected by numerous factors including the liquid used, the volume of the drop, ambient humidity and surface flatness. Studies by Baek et al. [42] and Li et al. [43] considered the effect of the external conditions on the measured contact angle in reverse osmosis (RO) membrane surfaces. It was established that using different droplet sizes, measuring times, liquid type and humidity can affect the contact angles of the material. However, there are no comparable studies involving polyamides (either filled or unfilled). In the case of polyamide 6, the measurement will be complicated because of the hygroscopic nature of the material [1], which can influence the accuracy of the contact angle measurement when using water. Moreover, the existence of fibres in fibre-reinforced polymer composites significantly increases the roughness of the material surface, which can also affect the contact angles. Therefore, it is clear that there is a need for systematic investigation of the contact angle measurement procedure on GFPA6 materials, including characterisation of the effect of variables, such as the liquids used, orientation used to measure the angle, the location on the sample surface and the volume of the droplets.

Hence, the aims of this study were two-fold. The primary aim was to investigate the effect of μPlasma modification on the wettability of GFPA6 surfaces, and their short-term (minutes to hours) and long-term (days to weeks) hydrophobic recovery. The secondary aim was to develop a more reliable systematic protocol for measuring contact angles on GFPA6 to better characterise the surface wettability and ageing behaviour after the treatment. Finally, approaches that will hinder or prevent complete hydrophobic recovery of the treated surfaces are briefly explored.

## 2. Materials and Methods

### 2.1. Material

Celstran^®^ CFR-TP PA6 GF60-03 tape (GFPA6, Celanese Corporation, Dallas, TX, USA), which is a polyamide-6 matrix (40 wt%) reinforced with continuous unidirectional glass fibre (60% by weight), was used for all testing within this study. Thermal analysis was carried out using a differential scanning calorimeter (PerkinElmer DSC-7, PerkinElmer Inc., Waltham, MA, USA), and the melting temperature (T_m_) and glass transition temperature (T_g_) were found to be 222.0 °C and 49.8 °C, respectively. The measured heat of fusion and calculated degree of crystallinity was found to be 43.88 Jg^−1^ and 19.08%, respectively.

### 2.2. μPlasma Modification

**Figure 1:**
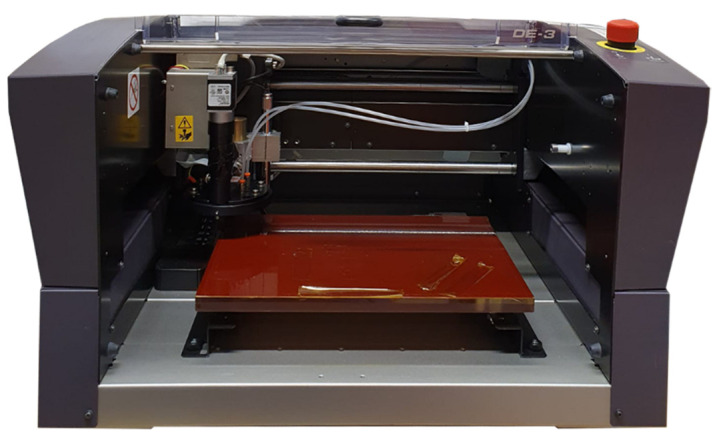
Image of μPlasma system.

**Figure 2:**
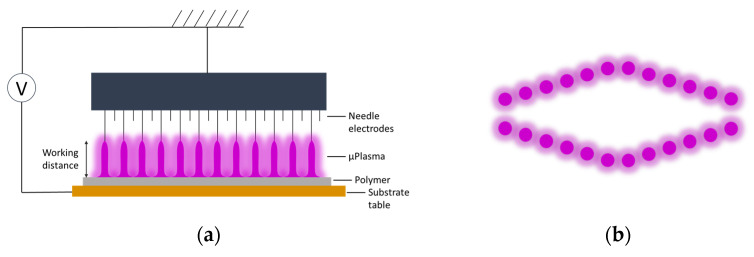
(**a**) Schematic of μPlasma modification setup. (**b**) μPlasma discharges pattern generated by 24 needle electrons from top view.

**Figure 3:**
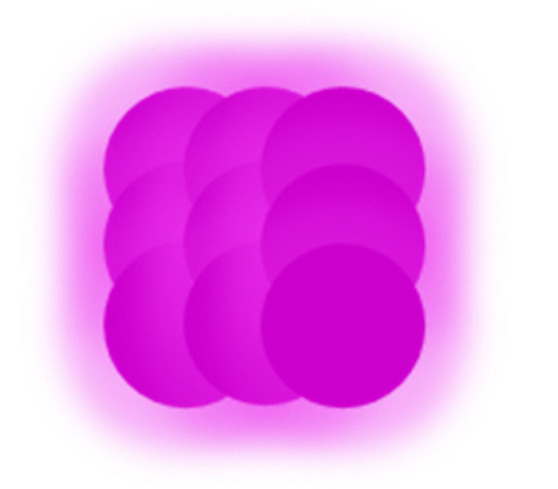
Plasma modification pattern.

The untreated GFPA6 was initially cleaned using ethanol to remove any contaminants on the surface, and then the surface was dried in air. The cleaned sample was then μPlasma treated using a Roth & Rau Pixdro LP50 plasma inkjet printer (InnoPhysics, Eindhoven, The Netherlands) (Figure 1) fitted with an InnoPhysics POD24 print-head (as illustrated in Figure 2a). There are 24 needles placed in two rows on a movable print head (as shown in Figure 2), which is connected to a high voltage alternating current power supply. The plasma within this study was ignited using a single row of 12 needles, and different combinations of parameters were tested, coupled with results of material wettability (i.e., contact angle measurements) to optimise the treatment parameters. The optimal settings were determined as follows: an accelerating voltage of 7 kV, a printing rate of 20 mm/s, and a working distance from the sample surface to the tips of the printing needles of 100 μm. Patterns were designed as 20 × 20 mm^2^ squares, where the plasma spots overlap with each other (shown in Figure 3), to produce uniform treatment coverage.

### 2.3. Scanning Electron Microscopy (SEM)

A JEOL 7000F SEM (JEOL, Tokyo, Japan) with an accelerating voltage of 5 kV was used to characterise changes in the surface morphology following treatment of the GFPA6 material. Before SEM imaging, samples were gold sputter-coated using an EMITECH K550 set (EMITECH, Kent, UK) at a current of 25 mA and for a duration of 2 min.

### 2.4. Contact Angle Testing

Contact angle measurements were carried out using the sessile drop method with an experimental apparatus that complied with the ISO 19403-2:2020 standard [44]. Samples were placed on a height (*z*-axis) adjustable laboratory jack, while a light source and a camera connected to a monitor were used to obtain a clear image of the droplet. The light source, sample surface and camera were placed in a straight line and at the same horizontal plane (i.e., same height/*z*-axis) to ensure contact angle measurements were obtained from the same viewpoint across different samples. Three repeats were performed on each sample to allow for average and standard deviation calculations of the measured contact angles. The measurements were carried out under ambient temperature and humidity conditions, where deionised water, diiodomethane, and glycerol were used as the liquids for the tests. To limit the potential influence of gravity, droplets were carefully deposited on the sample surface using a calibrated pipette. The average of the left and right contact angles was obtained for each individual droplet. For both wettability and ageing testing of sample surfaces, the overall average contact angle was calculated from the three repeat droplets. The captured images were analysed using the contact angle plugin on ImageJ to obtain contact angle values.

### 2.5. Wetting Envelope and Surface Free Energy

To better understand the wettability changes following the μPlasma modification, surface free energy values were calculated, producing wetting envelopes to evaluate the changes in the surface free energy and predict the wetting capabilities against different liquids. Surface free energies of the surfaces were calculated based on Young’s Equation (1), which describes the relationship between the surface energy of a solid (*σ_S_*), the surface tension of a liquid (*σ_L_*), the solid-liquid interface tension (*γ_SL_*), and the contact angle formed at the solid-liquid interface (*θ*), as well as Fowkes theory of splitting the total surface energies (both solid surfaces and liquids) into a combination of a dispersive, *σ^D^*, and a polar, *σ^P^*, component. Equation (3), which was used to calculate the surface energies, forms from the combination of Equations (1) and (2).

(1) $$\sigma_{S}=\gamma_{SL}+\sigma_{L}cos\theta$$

(2) $$\gamma_{SL}=\sigma_{S}+\sigma_{L}-2\cdot \left(\sqrt{\sigma_{S}^{D}\cdot \sigma_{L}^{D}}+\sqrt{\sigma_{S}^{P}\cdot \sigma_{L}^{P}}\right)$$

(3) $${\left(\sigma_{L}^{P}\right)}^{1/2}\cdot {\left(\sigma_{S}^{P}\right)}^{1/2}+{\left(\sigma_{L}^{D}\right)}^{1/2}\cdot {\left(\sigma_{S}^{D}\right)}^{1/2}=\frac{\sigma_{L\;}\;\left(cos\theta +1\right)}{2}$$

where *σ_L_^P^* is a polar component of the liquid surface tension, *σ_S_^P^* is the polar component of solid surface energy, *σ_L_^D^* is a dispersive component of the liquid surface tension, *σ_S_^D^* is the dispersive component of solid surface energy, and *σ_L_* is the total liquid surface tension. The dispersive and polar components of solid surface energy, *σ_S_^P^*, were calculated by applying two different liquids, diiodomethane (*σ_L_^D^* = 50.8 mN/m, *σ_L_^P^* = 0) and deionised water (*σ_L_^D^* = 46.4 mN/m, *σ_L_^P^* = 26.4 mN/m) to Equation (3). Therefore, the total surface energy of the solid, *σ_S_*, was calculated as *σ_S_* = *σ_S_^P^* + *σ_S_^D^*.

When the polar and dispersive components of a standard liquid are brought into a coordinate system as a function, a wetting parameter *R* can be obtained using Equation (4), with polar (*R·cosϕ*) and dispersive (*R·sinϕ*) component contributions to the total magnitude of the surface energy being calculated using the formed angle (*ϕ*).

(4) $$R^{2\;}={\left(\sigma_{L}^{P}\right)}^{2}+{\left(\sigma_{L}^{D}\right)}^{2}$$

(5) $$\sigma_{L}^{P}=R\cdot cos\phi$$

(6) $$\sigma_{L}^{D}=R\cdot sin\phi$$

Substituting these Equations (4)–(6) into Equation (7), which describes the surface energy contributions for a solid and liquid under complete wetting (i.e., *cosθ* = 1 or contact angle = 0), forms Equation (7). In this equation, R, which is the absolute vector, can be calculated for different *ϕ* angles between 0 and 90°, to form a wetting envelope that describes the polar and dispersive surface tension limits for theoretical complete wetting of the surface. Comparisons of the area within the produced wetting envelope can give information related to the wetting capabilities of a surface, with larger wetting envelopes indicating greater surface wettability (i.e., more liquids are expected to be capable of fully wetting the surface).

(7) $$\;\;R={\left(\frac{\sqrt{\sigma_{s}^{P}}sin\phi +\sqrt{\sigma_{s}^{D}cos\phi }}{cos\phi +sin\phi }\right)}^{2}\;for\;0^{\circ }\leq \phi \leq 90^{\circ }$$

## 3. Results and Discussion

### 3.1. μPlasma Modification Process Optimisation

**Figure 4:**
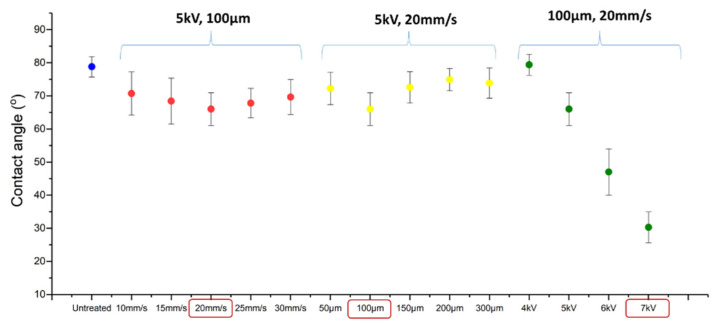
Contact angles of an untreated sample and μPlasma treated samples with different processing parameters (printing rate, working distance and accelerating voltage).

To optimise the μPlasma modification settings, the contact angles of untreated and μPlasma treated samples under different settings, including printing rate, the working distance (from the sample surface to the tips of the printing needles) and accelerating voltage, were measured (as shown in Figure 4). When keeping the voltage and working distance constant, but changing the printing rate, the contact angles do not demonstrate substantial change. Although not significantly different, the biggest reduction in contact angle was found under 20 mm/s printing rate, and therefore, this was chosen for the following optimisation tests. When the voltage and printing rate were kept constant and the working distance was adjusted, the biggest reduction of contact angle was observed with a working distance of 100 μm. Therefore, a working distance of 100 μm (and printing rate of 20 mm/s) was chosen for the final process optimisation test (influence of accelerating voltage). A trend of continuously reducing contact angles was observed with increasing accelerating voltage, with an accelerating voltage of 7 kV demonstrating the largest reduction. This reduction in water contact angles was measured to be significant, going from 78.8 ± 3.1° (untreated) to 32.5 ± 4.7° following μPlasma modification at 7 kV. This indicates that the accelerating voltage is the dominating parameter of the μPlasma modification process for controlling final sample surface wettability. A higher accelerating voltage of 8 kV was also attempted; however, issues with the stability of the plasma prevented the further use of this setting (plasma remained active in between treatment scan lines).

**Figure 5:**
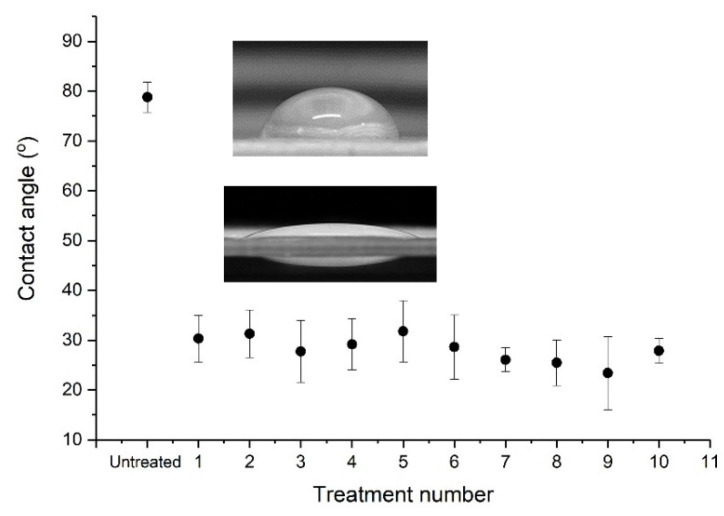
Contact angles of untreated and treated GFPA6 from 1–10 treatments.

The increase in wettability can be attributed to the increase of the total surface energy and the formation of new polar groups following μPlasma modification (a more detailed explanation can be found in Section 3.4). The contact angles of untreated and μPlasma treated samples (1–10 treatment scans) are displayed in Figure 5. It was observed that no discernible enhancement or synergetic improvement could be observed in the surface wettability with increasing treatment numbers, i.e., it was found that a single treatment scan on the sample surface was sufficient to modify the wettability to the same degree as multiple scans. Similar results have been previously reported by Károly et al. [45], whereby no significant further enhancement in wettability of unfilled polyamide 6 surfaces was found with increasing plasma treatment time. Therefore, since increasing treatment numbers did not significantly enhance the wettability of the surfaces when compared to single treatment scans, all other tests performed within this study were carried out with surfaces subjected to a single treatment scan. In summary, one treatment was found to induce enough modification of the surface to approximately reduce the contact angle by 50°.

### 3.2. Ageing of GFPA6 Surface after μPlasma Modification

**Figure 6:**
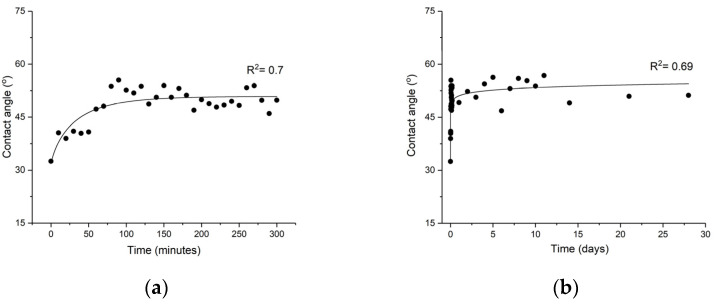
Change of contact angles with ageing following μPlasma modification for: (**a**) 5 h, (**b**) 4 weeks.

Although μPlasma modification can significantly improve the wettability of GFPA6 surfaces, the effect was found to not be permanent when the sample surface was exposed to air. A sharp increase in the measured contact angles was observed immediately following μPlasma modification (0–80 min), revealing a fast ageing mechanism. Figure 6a shows the increase in surface contact angle of μPlasma treated GFPA6 samples left to age in the air for 5 h. The value of the measured contact angle rose from 32.5° to 40.6° within the first 10 min, with a further increase to 53.7° after 80 min. This decrease in hydrophilicity is known as hydrophobic recovery, which is typically attributed to the reorientation and diffusion of the polar functional groups that are produced, and introduced onto the surface of the samples, during the μPlasma modification. The increase in contact angles is much less significant between 80 min and 3 h, with gradual plateauing of the measured contact angles. Despite the ageing process, the measured contact angle did not return to values comparable to untreated samples; the wettability modification of the sample surface partially remained, even up to 4 weeks of ageing (as shown in Figure 6b). This may develop due to the interaction between the polar groups formed on the GFPA6 surface and the water molecules on the top surface, with the absorption of moisture from the air acting to provide a polar environment that protects the decay of the polar groups. In summary, the μPlasma treated sample age and loses much of the wettability introduced onto their surfaces within the first two hours following treatment, and then levelled off in the next 4 weeks.

### 3.3. Optimisation of the Contact Angle Measurement Procedure

#### 3.3.1. Optimisation of the Contact Angle Measurement Protocol

**Figure 7:**
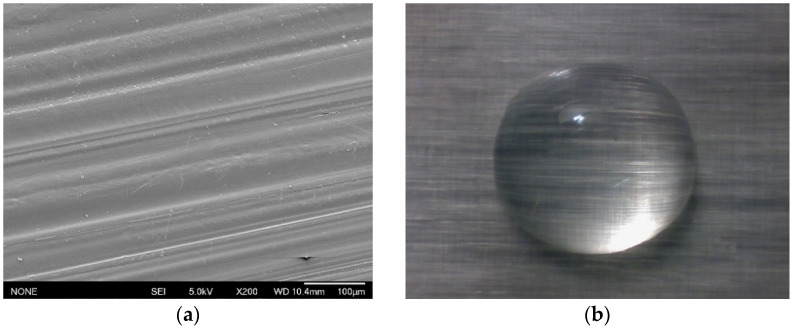
(**a**) SEM image of GFPA6 surface morphology, and (**b**) image of GFPA6 surface covered with a 6 μL water droplet.

**Figure 8:**
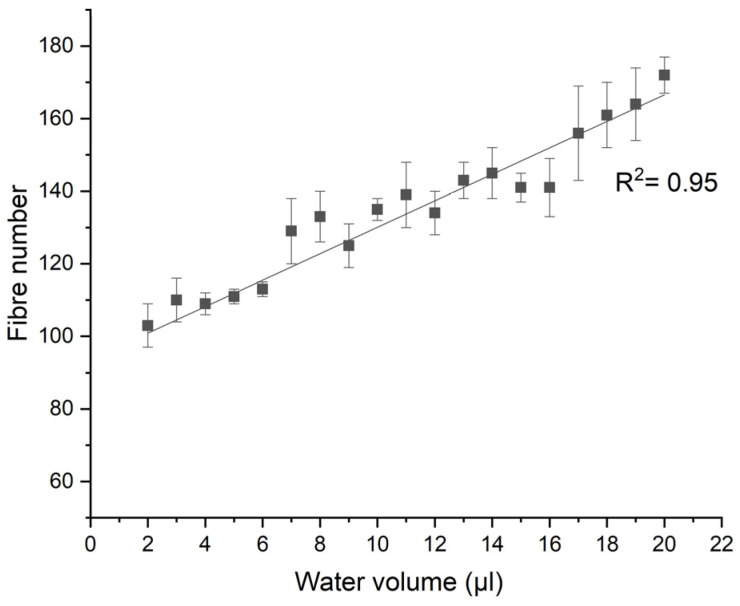
Graph displaying the effect of water droplet volume on the number of fibres covered on the untreated sample surface.

The glass fibre reinforcement was clearly visible in the composite samples, and this is illustrated in Figure 7a. To ensure that a sufficiently large and representative surface area was covered by the water droplet, the contact angle was recorded as a function of droplet size in the range of 2 to 20 μL. The number of fibres covered by each droplet was measured using ImageJ from SEM images, as shown in Figure 7a. Figure 8 shows the calculated number of fibres covered by each droplet size. The number of the fibres covered by the droplet increases with the droplet size. A 2 μL droplet has been calculated to cover approximately 103 fibres, which is assumed to be sufficient to reduce the contact angle error associated with the deviations introduced due to the fibre protrusions on the surface. Since all tested droplet sizes were found to be sufficient, 6 μL droplets were used within this study as they also correspond to the ISO standard (ISO 19403-2:2020), which enables clearer and more accurate droplet contours to be mapped during software angle measurements.

**Figure 9:**
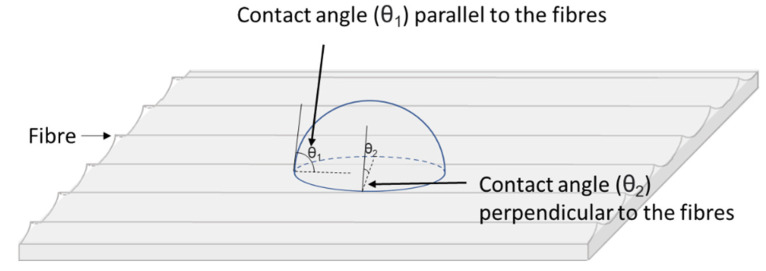
Schematic diagram of water contact angles measured parallel or perpendicular to the orientation of the fibres.

**Figure 10:**
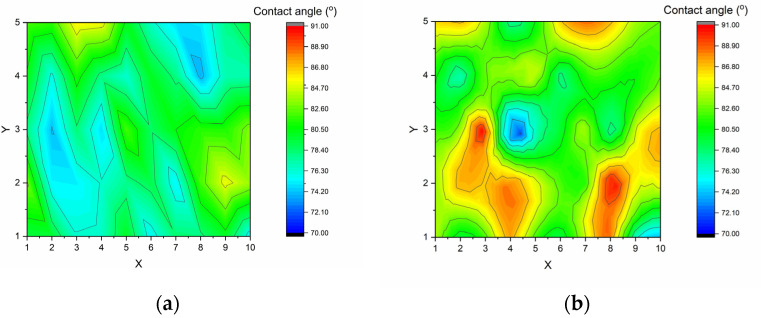
Contact angles distribution represented by different colours on the untreated sample (5 × 10 cm^2^) when measured at different fibre orientations: (**a**) parallel, (**b**) perpendicular to the fibres.

As shown in Figure 5a, two observer directions are possible when measuring contact angles, either parallel or perpendicular to the fibres, with the alignment of the fibres potentially influencing the measured value of the contact angles (as illustrated schematically in Figure 9). Therefore, contact angle mapping of the surfaces was performed using 50 deionised water droplets produced on a 5 × 10 cm^2^ untreated sample surface, with measurements being recorded from both a parallel (a) and perpendicular (b) perspective to the fibres. The distribution of the contact angles of the 50 droplets are represented using colour heat mapping in Figure 10. The result demonstrates that the standard deviation is larger when the contact angles are measured perpendicular (±4.3°), rather than parallel (±3.1°), to the fibres. It can be seen that the colour distribution is more uniform when measuring parallel to the fibres, this difference may arise due to the superficial fibres present in the perpendicular direction acting as walls to impede the spread of the droplet. This can, therefore, promote increased cohesion of the water molecules, thereby forming larger angles against the surface, or alternatively, when the force of water droplet is great enough to break beyond the wall, the angle returns to normal, or even smaller. Ultimately, this results in the incorrect measurement of contact angles, and increased variation in measured data, in comparison with measurements parallel to the fibres. Hence, all other contact angles measurements in this experiment were recorded parallel to the fibres, to enable accurate and reliable data to be recorded for the fibre reinforced polymer surface.

#### 3.3.2. Optimisation of the Wettability Ageing of GFPA6 Samples

**Figure 11:**
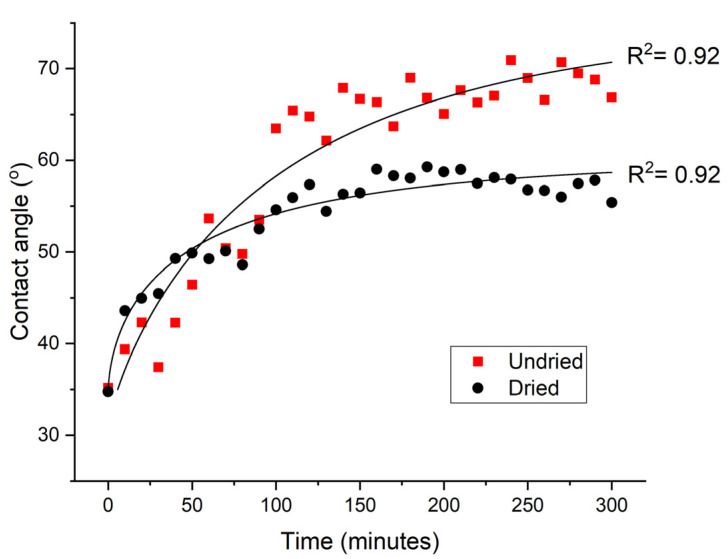
Ageing of dried and undried GFPA6 surface represented by contact angles after μPlasma modification.

Given that polyamide 6 is hydrophilic, the water content in the sample may influence the ageing behaviour. To investigate this, the post-treatment time-dependence of the contact angles of untreated material was determined. Samples were either stored in the air prior to treatment or had been oven dried for 3 h, contact angles were then recorded every 10 min for 5 h following μPlasma modification. It was observed that when the sample was dried before the treatment, the ageing process was impeded (Figure 11). The measured contact angle was found to plateau at 59.3° after 5 h of ageing, while the undried sample reached a plateau at 71.2°, with signs of further ageing still in progress. This suggests that the pre-existing moisture in the untreated material plays a significant role in facilitating the ageing process. This can potentially be attributed to the hydrated material providing a polar environment for the polar groups generated by the μPlasma modification, therefore, facilitating the reorientation process of the polar groups and diffusion back into the bulk, which results in a less polar surface. Additionally, the moisture is mostly absorbed in the amorphous phase of the polymer, which can induce a reorganisation of the hydrogen bond structure of the material, leading to an expansion of the free volume and increased mobility of surface chains. Therefore, drying of the samples prior to μPlasma modification is necessary to slow down the ageing process.

**Figure 12:**
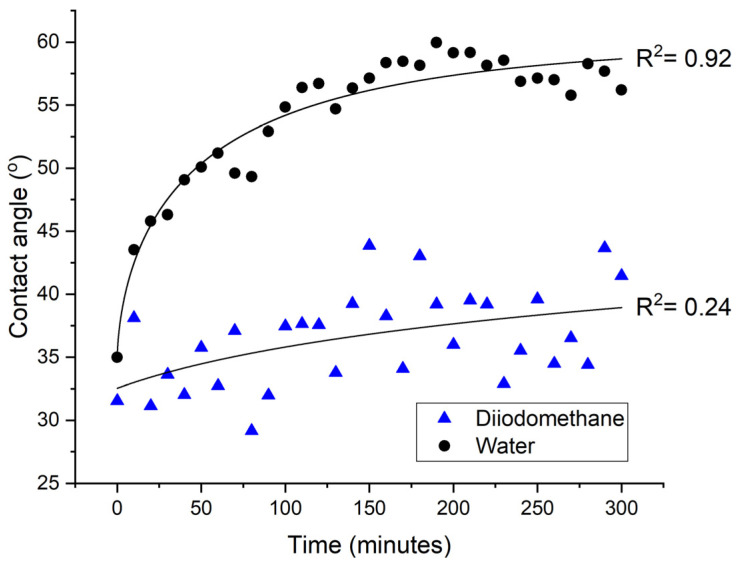
Ageing of GFPA6 surface represented by water and diiodomethane contact angles on the same locations after μPlasma modification.

**Figure 13:**
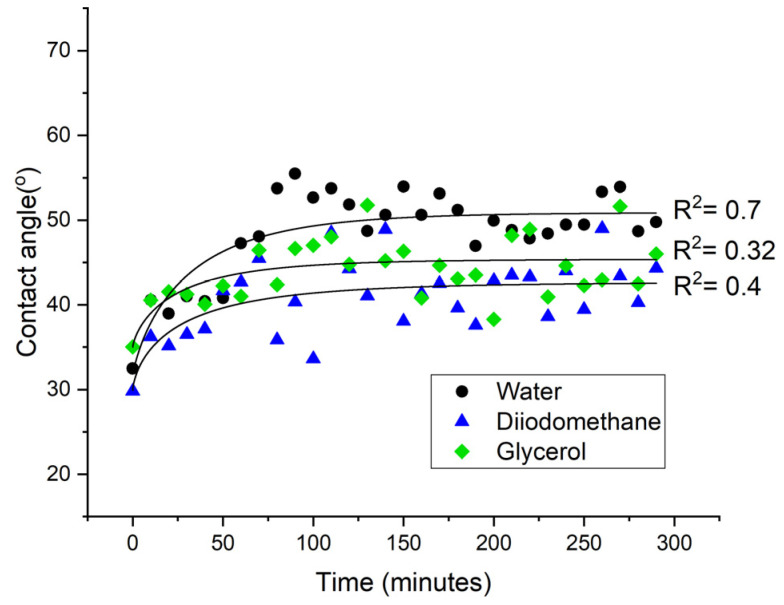
Ageing of GFPA6 surface represented by water, glycerol and diiodomethane contact angles on different locations after μPlasma modification.

As the μPlasma modification is highly sensitive to the working distance (distance between needle tips to sample surface), the roughness caused by the fibres in the surface of the material can affect the uniformity of the μPlasma modification, and therefore, the resulting contact angle tests are prone to fluctuations. To optimise the measurement, and eliminate the inconsistencies, measurements of the contact angles on the same location are potentially more reliable. However, since the material is hydrophilic, it can absorb water during the measurements process, and this will influence the spreading of subsequent droplets on the surface (and also promote the ageing process as described above). Therefore, a different liquid diiodomethane, which is not absorbed by the material and vaporises quickly in the air, was used for contact angle tests on the same locations. It can be seen in Figure 12 that although the contact angles measured using diiodomethane show a similar trend to those made using water, large fluctuations compared to water measurements are observed and this masks the early stages of the ageing process. Therefore, it is not feasible to test contact angles on the same sample location using either water or diiodomethane, and different sample locations must be used for subsequent contact angle measurements. Results of contact angles of different liquids including water, diiodomethane and glycerol are shown in Figure 13. Among them, deionised water was found to give relatively lower fluctuations.

In summary, there are two features of the GFPA6 material that need to be considered during the contact angle measurements for GFPA6, the absorbance of water into the matrix and the roughness caused by fibres on the surface. Using water droplets as small as 2 μL is sufficient to cover enough fibres to reduce errors. In this study, we used 6 μL, as recommended in the ISO standard, for increased consistency during the testing. The water can facilitate the ageing process; therefore, the samples need to be dried before the treatment, and testing on the same locations should be avoided. For a hydrophilic material, diiodomethane is more suitable for testing on the same locations; however, there is far more variability in comparison to water.

### 3.4. Wetting Envelope and Surface Energy

**Figure 14:**
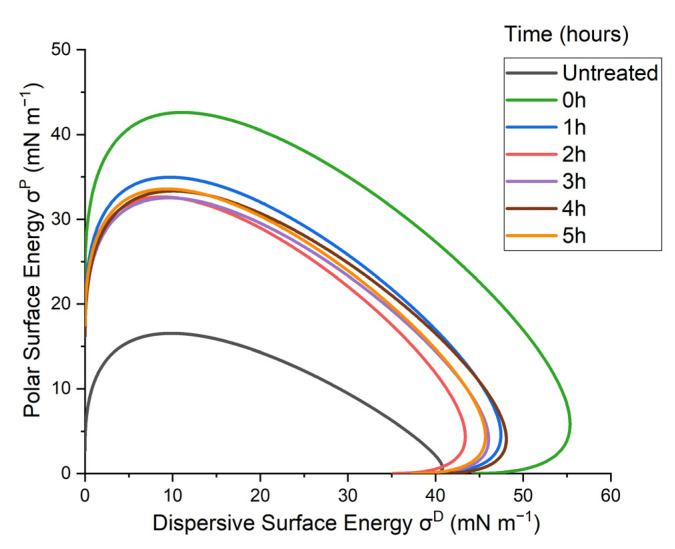
Wetting envelopes of untreated GFPA6 surface and μPlasma treated as a function of time (0–5 h after the treatment).

The wetting envelope (shown in Figure 14) can be used to extrapolate the wettability of the untreated and treated surfaces, to hypothetical liquids with varying surface free energies, using the calculated surface free energies obtained with the Fowkes equation. When the polar (*σ^S^*) and dispersive components (*σ^D^*) of a liquid are within the enclosed area of the produced wetting envelope, complete wetting (i.e., *cosθ* = 1 or contact angle = 0°) of the surface can be expected. As shown in Figure 14, the total area of the wetting envelope was the smallest for the untreated sample, before significantly increasing following the μPlasma modification (at 0 h). This indicates a significant enhancement of the overall surface wettability of the GFPA6 following the initial treatment of the surface. The total area of the wetting envelope was then found to have a sharp, but smaller, decrease after one hour of ageing, with minimal changes over the following 2–5 h, therefore, indicating relatively stable surface wettability. However, it should be noted that, in all cases, the surface wettability of the treated surfaces was found to increase in comparison to the untreated surfaces.

**Figure 15:**
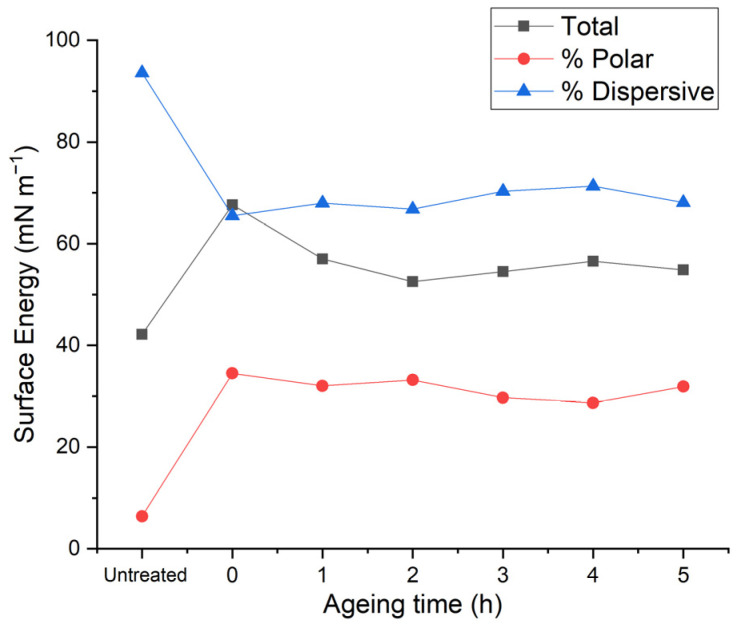
Total, polar and dispersive surface energy of untreated and treated GFPA6 ageing.

To better understand the wettability, the surface energy of untreated GFPA6 and treated GFPA6 aged for 5 h were measured using two different liquids (deionised water and diiodomethane). It can be seen from Figure 15 that the total surface free energy increased from 42.2 mN/m to 67.6 mN/m. This increase can be attributed to the change in the polar component of the surface free energy, which significantly increased from 6.4 mN/m to 34.5 mN/m, and the decrease of the dispersive component of the surface free energy, from 93.6 mN/m to 65.5 mN/m. The total surface free energy decreased by 10.6 mN/m one hour after the treatment, with the polar and dispersive components decreasing and increasing by 2.5 mN/m each, respectively, therefore, indicating a slight reduction in surface wettability. All three surface free energies values (total, dispersive component, polar component) plateaued in the following three hours, remaining significantly higher than the total surface free energy of the untreated surfaces. This demonstrates that the μPlasma modification can both, significantly enhance the surface wettability, and retain elevated wettability following 5 h of ageing.

The results of the surface energy were consistent with the wetting envelope. The increase of wetting property of the GFPA6 right after the μPlasma modification was due to the increase of polar groups produced by the plasma. Although the treated sample recovered its property fast even in the first one hour, the wetting property was still partially remained when the surface energy levelled off over the next three hours.

### 3.5. Storage of Treated GFPA6

**Figure 16:**
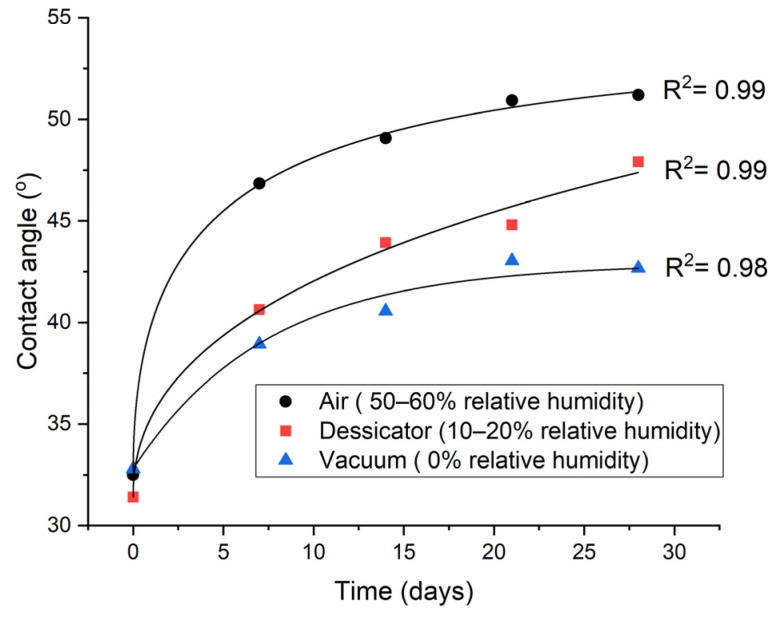
Ageing when store the μPlasma treated GFPA6 at different conditions.

Figure 16 shows the variation of the water contact angle of μPlasma treated GFPA6 surfaces stored under different conditions in the air (with a humidity of 50–60%), in a desiccator (with humidity of 10–20%), and under vacuum for 4 weeks (with assumed humidity of 0%). It can be seen that all samples experienced hydrophobic recovery, but to varying degrees under different conditions. The ageing process was faster when the samples were stored in higher humidity environments. This could be due to the higher humidity causing plasticisation of the polymer, thus decreasing the glass transition temperature [46] and leading to an increased free volume on the surface, and higher mobility of the polymer chains at, or near, the surface, which accelerated hydrophobic recovery [47]. Furthermore, the hydrophilic groups generated by plasma may have been partially dissolved by water that was absorbed into the material from the environment. It was observed that the ageing process was slowed down significantly when the sample was stored in the vacuum, which suggests water was not the only variable facilitating ageing, but also the air. This could be due to the radicals that are produced by plasma treatment being trapped on or near to the material surface, which can then continuously react with the oxygen in the air after the treatment [40]. Therefore, storing the μPlasma treated samples appropriately can largely inhibit the ageing process after μPlasma modification.

## 4. Conclusions

This study has shown that the novel technology of μPlasma modification can effectively improve the wettability of the GFPA6 surfaces and benefits are achieved after only a single treatment scan (water contact angles decreased from 78.8° to 32.5°). However, hydrophobic recovery is observed (contact angle recovered to 53.7° after 80 min) and there is a lack of permanence in these benefits. It was apparent that the sample environment strongly influenced the timescale and extent to which hydrophobic recovery could take place with dry atmospheres and dried samples clearly hindering the recovery process. The study has also shown that it is more reliable to measure contact angles on different locations of the surface due to the hydrophilic nature of the material.

The contact angle measurements were optimised for the untreated GFPA6 surfaces and for representing the ageing behaviour after the μPlasma modification. Regarding the increase of roughness on the surface caused by the fibres, even with 2 μL it was found to sufficiently minimise the error caused by the varying fibre coverage. The orientation of the fibres was found to affect the accuracy of the contact angle measurements, with measurements parallel to the fibres demonstrating higher consistency compared to measurements perpendicular to the fibres. High surface roughness influences the uniformity of the μPlasma modification due to the high sensitivity of the device to the working distance between the needles and the specimen surface. As it is not absorbed by the material, evaporates quickly in air, and does not facilitate the accelerated ageing of the surface, diiodomethane was used to test on the same location of the sample surface to reduce the inconsistency of the contact angle measurements. However, the result demonstrated that the diiodomethane contact angles fluctuated greatly, making it difficult to see clear trends of the ageing behaviour. Consequently, contact angle testing on the different locations was found to give relatively more accurate results. Different liquids were used to test ageing on the treated surface of different locations. All three liquids tested (deionised water, diiodomethane and glycerol) showed similar trends of ageing. Among them, deionised water was found to give relatively lower fluctuations. Therefore, deionised water on different locations on the μPlasma treated GFPA6 surface was deemed the most appropriate approach. The results of contact angles of deionised water and diiodomethane were used to calculate the surface free energies of the samples and to plot their wetting envelopes. The change of surface free energy was consistent with the wetting envelope. The increase of wettability of the GFPA6 was attributed to the relative increase in the polar component content following μPlasma modification. Although the treated samples initially recovered their total surface free energy within the first hour, the wetting properties were still partially retained over the next 4 weeks.

## Data Availability

Data is contained within the article.
